# Simulated Impact of Vitamin A‐Fortified Sugar on Dietary Adequacy and Association of Usual Sugar Intake With Plasma and Breast Milk Retinol Among Lactating Zambian Women

**DOI:** 10.1111/mcn.70077

**Published:** 2025-08-06

**Authors:** Demewoz Haile, Reina Engle‐Stone, Bess Caswell, Hanqi Luo, Kevin W. Dodd, Charles D. Arnold, Modou Jobarteh, Matthew Greene, Mackford Chipili, Marjorie J. Haskell, Amanda C. Palmer

**Affiliations:** ^1^ Institute for Health Metrics and Evaluation University of Washington Seattle Washington USA; ^2^ Institute for Global Nutrition University of California, Davis Davis California USA; ^3^ Western Human Nutrition Research Center, Agricultural Research Service US Department of Agriculture Davis California USA; ^4^ Hubert Department of Global Health, Rollings School of Public Health Emory University Atlanta Georgia USA; ^5^ National Cancer Institute National Institutes of Health Bethesda Maryland USA; ^6^ Imperial College London London UK; ^7^ Department of International Health Johns Hopkins Bloomberg School of Public Health Baltimore Maryland USA; ^8^ District Medical Office for Mkushi District Mkushi Zambia; ^9^ The Johns Hopkins Bloomberg School of Public Health Center for Human Nutrition Baltimore Maryland USA

**Keywords:** dietary modelling, fortification, lactating women, vitamin A

## Abstract

In Zambia, mandatory sugar fortification with vitamin A (VA) has been implemented, but its impact on VA inadequacy and status has yet to be assessed. This study evaluated the contribution of VA‐fortified sugar to dietary VA adequacy and the relationship between dietary intakes and VA status in 243 lactating women, based on 24‐h dietary recalls in Mkushi, Zambia. We estimated usual intake distributions and the prevalence of VA adequacy using the National Cancer Institute (NCI) method across five scenarios: without sugar fortification; with fortification at 3.1 or 8.8 mg/kg (median levels previously measured in Mkushi); at 10 mg/kg (the minimum legal requirement at the household level), and at 15 mg/kg (the minimum legal requirement at the factory level). We applied the regression calibration method to examine associations of usual intake of sugar and dietary VA with plasma and breast milk retinol concentrations. Without fortified sugar, the estimated prevalence of dietary VA inadequacy was 83% (standard error [SE]: 6). Projected reductions in VA inadequacy were 7 (SE: 6), 24 (SE: 14), 30 (SE: 15) and 47 (SE: 18) percentage points for sugar fortification at 3.1, 8.8, 10 and 15 mg/kg, respectively. Usual sugar intake was not significantly associated with plasma or breast milk retinol concentrations. The potential impacts of sugar fortification on VA intakes are limited if the programme is not implemented as planned. Even if the target fortification levels are achieved (10 mg/kg), sugar fortification alone is unlikely to eliminate dietary VA inadequacy among lactating women in Zambia.

## Summary

- Large‐scale food fortification programmes are cost‐effective strategies to address micronutrient deficiencies. Zambia has been implementing a mandatory sugar fortification programme with vitamin A.
- Without accounting for fortified sugar, the estimated prevalence of dietary vitamin A inadequacy is alarmingly high among lactating women in Zambia. Fortifying sugar at and above the minimum legal requirement (10 mg/kg) could significantly reduce vitamin A inadequacy.
- Other complementary vitamin A interventions, in addition to strong monitoring of the table sugar fortification programme, are likely required to reduce vitamin A deficiency among lactating women in Zambia.

## Graphical abstract

In Zambia, mandatory sugar fortification with vitamin A has been implemented, but its impact on vitamin A intake and status is uncertain. Modelling of dietary data from lactating women suggests that the potential impacts of sugar fortification on VA intakes are limited if the programme is not implemented as planned.

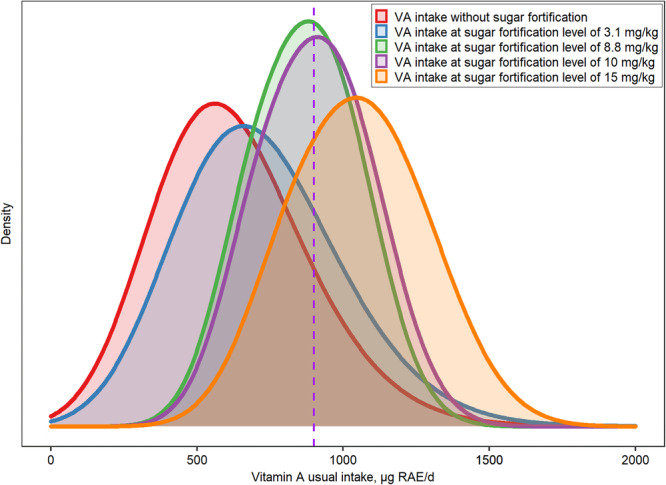

**Abbreviations**
24‐h: 24‐hour
AGP: α_1_‐acid glycoprotein
BMI: body mass index
BMR: breast milk retinol
CI: confidence interval
CRP: C‐reactive protein
EAR: estimated average requirement
HPLC: high‐performance liquid chromatography
LMICs: low‐ and middle‐income countries
LSFF: large‐scale food fortification
NCI: National Cancer Institute
P25: 25th percentile
P75: 75th percentile
RAE: retinol activity equivalent
SE: standard error
SES: socio‐economic status
UL: tolerable upper intake level
USDA: United States Department of Agriculture
VA: vitamin A

## 1 Introduction

Lactating women have the highest vitamin A requirement among various demographic groups due to the need to support secretion of adequate vitamin A for their infants in breast milk (US Institute of Medicine 2001). The vitamin A status of the mother during lactation is correlated with the breast milk vitamin A concentration and the vitamin A status of their breastfed infants (Da Silva et al. 2019). Lactating women who are unable to maintain breast milk concentrations greater than 1 μmol/L may be unable to provide sufficient vitamin A to build up the vitamin A stores of their breastfed infants (Stoltzfus and Underwood 1995). Thus, sufficient vitamin A intake during lactation is critical for meeting the dietary requirements of their infants and for the accumulation of liver stores needed after weaning (Cabezuelo et al. 2019; J. S. Ross and Harvey 2003; Stoltzfus and Underwood 1995).

In low‐ and middle‐income countries (LMICs), where intake of animal‐source foods is low, dietary provitamin A carotenoids are the main source of vitamin A (Haskell 2012). The bioavailability of provitamin A carotenoids is low compared to animal‐source foods, which makes it challenging for lactating women living in LMICs to meet their high vitamin A requirement (Arimond et al. 2018; de Pee and W. Bloem 2007; Sommer and Vyas 2012). Several studies from LMICs, including Zambia, reported a high prevalence of inadequate vitamin A intake among lactating women (Alaofe et al. 2014; Da Silva et al. 2019; Harika et al. 2017; Kaliwile et al. 2019; Wessells et al. 2019). Large‐scale food fortification programmes (LSFFs) have been identified as cost‐effective strategies to increase vitamin A intake and thus minimise the consequences of vitamin A deficiency (Bruins and Kraemer 2013; Fiedler and Macdonald 2009; Horton 2006; Phillips et al. 1996). A recent systematic review and meta‐analysis found that LSFF was associated with a reduction of vitamin A deficiency among children and women (Keats et al. 2019). Given the expected effectiveness and cost‐effectiveness of this strategy, fortification of edible oil and sugar with preformed vitamin A (in the form of retinol) has been implemented in several LMICs (Dary and Mora 2002; Mkambula et al. 2020). Industrial‐scale sugar fortification has been successful in addressing vitamin A deficiency in Central America (Martorell and de Romaña 2017). For example, introduction of vitamin A‐fortified sugar in Guatemala was associated with a reduction in prevalence of vitamin A deficiency (Martorell and de Romaña 2017), and the vitamin A contribution from fortified sugar is a major determinant of reaching dietary adequacy among pregnant and lactating women (Bielderman et al. 2016). In Guatemala, sugar fortification significantly increased retinol concentration and decreased the prevalence of breast milk retinol levels below 20 µg/dL by 50% after a year of implementation (Pineda 1998). In Indonesia, edible oil fortification was associated with increases in breast milk and plasma retinol (Sandjaja et al. 2015).

Zambia was the first country in sub‐Saharan Africa to implement mandatory industrial‐scale sugar fortification with vitamin A in 1998, and the programme has continued since then (Sablah et al. 2014). However, having a fortification programme in place does not guarantee an impact. The impact of a fortification programme depends on various factors such as reach of the fortified foods, fortification level and magnitude of the inadequacy gap of the target nutrient among the vulnerable group (Fiedler et al. 2013; Osendarp et al. 2018). In Zambia, studies have shown that sugar fortification levels might not always adhere to the target levels (Greene et al. 2017; Hotz et al. 2012). Thus, there is some uncertainty regarding the potential impact of Zambia's sugar fortification programme on dietary adequacy and vitamin A status. Concerns have also been raised that vitamin A intakes from fortified sugar may be contributing to excessive intakes (Tanumihardjo et al. 2015, 2019). The contribution of vitamin A‐fortified sugar to the adequacy of vitamin A intake among lactating women has not been evaluated in Zambia. This study had the following objectives: (i) Predict the impact of sugar fortification with various levels of vitamin A on the prevalence of vitamin A intake below the estimated average requirement (EAR) and retinol intake above the tolerable upper intake level (UL) and (ii) to examine the association between sugar intake and vitamin A status biomarkers, accounting for usual vitamin A intake from other foods among lactating women.

## 2 Methods

### 2.1 Study Setting and Design

This analysis used data from the baseline assessment of 243 lactating women who participated in a community‐based clinical trial designed to evaluate the impact of industrially fortified maize or provitamin A carotenoid‐biofortified ‘orange’ maize consumption on vitamin A status of infants and lactating women in Mkushi, a town in Zambia's Central Province (Palmer et al. 2021). Previous research from this area indicated a high prevalence of vitamin A deficiency and other forms of undernutrition (Hotz et al. 2011; Palmer et al. 2016). The original trial identified eligible study participants in three steps. First, all lactating women who attended under‐five clinic visits with their infants at Mkushi District Hospital between July 2015 and September 2016 were registered.

Then, a subset of the registered lactating women from the highest population‐density neighbourhoods were consented and enrolled into the surveillance cohort. Finally, all women in the surveillance cohort were visited at their homes 1 week before the infants reached 9 months. This visit was to obtain informed consent for further screening procedures and to conduct a final check of eligibility for the trial. From the eligible women, detailed socio‐demographic, morbidity and maternal dietary data (described below) were collected at 9 months postpartum. Four days later, the dietary recall was repeated. At 9 months and 10 days, a third round of dietary data and biological samples (whole blood and full breast milk) were collected. Additionally, maternal height (Shorrboard portable stadiometer) and weight (Seca 874 digital scale) were measured using standard protocols. The detailed lab analysis and quality control procedure of the biological samples were described elsewhere (Palmer et al. 2021).

### 2.2 Dietary Data Collection

Three rounds of dietary intake data were collected from lactating women using a tablet‐based 24‐h recall. The tablet‐based 24‐h recall tool used a modified multiple‐pass method, first prompting the respondent to describe foods as they were consumed sequentially through the day. All food description details, including added ingredients and portion size, were collected for each recalled food item. A detailed description of the tablet‐based 24‐h recall tool used in this study has been published elsewhere (Caswell et al. 2015; Caswell et al. 2018).

The days of the week on which to collect dietary intake were randomly assigned to get representation of all days of the week except Saturday. The study participants were asked to recall all foods and drinks that they consumed between waking the previous day and waking the day of the interview. For most foods, the respondents were asked to describe the amount consumed by indicating the closest match among five portions of a similar food shown in a photo booklet. The photo booklets were developed based on the procedure as described in Caswell et al. (2015). We recruited 12 women to guide the development of the portion sizes. We asked each of the women to serve typically prepared foods from a local restaurant onto a plate in the amounts perceived by each woman as ‘small’, ‘medium’ and ‘large’. We weighed the portions and photographed five serving sizes representing the minimum portion, median small, medium, large portions and maximum portion. The portion weights were estimated based on the weight of the portion size photograph from the 24‐h recall and adjusted for density. For other foods, respondents were asked to report the number of food units consumed. For recipes or mixed dishes, information was collected on the types and amounts of ingredients, cooking methods, and the total amount of the recipe prepared.

### 2.3 Quantification of Observed Nutrient Intake

For this analysis, a local food composition table was compiled, primarily based on the food composition table developed and provided by HarvestPlus for Zambia (HarvestPlus, unpublished results; National Food and Nutrition Commission 2007b). Whenever food composition data were not available for a specific food within the HarvestPlus for Zambia food composition table, data from other food composition tables and United States Department of Agriculture (USDA) Nutrient database were used (Agricultural Research Service & US Department of Agriculture 2014; Hotz et al. 2012; Korkalo et al. 2011; National Food and Nutrition Commission 2007a). Additional recipe information was collected through focus group discussions with local women. A set of standardised recipes for Zambian mixed or prepared foods by local women was the primary source of data on ingredient proportions for mixed or prepared food. Nutrient contents of foods were calculated by multiplying ingredient or unmixed food weights by nutrient contents from the food composition table. When necessary, the nutrient content of the cooked food was imputed from the raw food adjusted using nutrient retention factors published by USDA. The total nutrient (total vitamin A and retinol) intake of an individual was the sum of the entire nutrient intake derived from all foods consumed over a day. Total vitamin A intake was expressed as micrograms retinol activity equivalents (RAEs). We also calculated total preformed vitamin A intake for assessment of the risk of retinol intake above UL.

We had 690 person‐days of dietary intake data from the three repeated 24‐h dietary recalls. We excluded 10 person‐days of data with energy intake higher than mean + 3 SD (mean = 2421 kcal/day, SD = 1037 kcal/day, mean + 3 SD = 5532 kcal/day) from the dietary analysis. Energy intake reports that fall beyond + 3 SD of the mean are more likely to be outliers and can indicate a substantial measurement error. Including observation with implausible energy intake could skew usual intake distribution and distort the association between dietary intake and health outcomes, as energy intake is correlated with both macronutrient and micronutrient intake.

The average reported energy intake to predicted basal metabolic rate ratio was 1.72, falling within the expected range according to the Goldberg criterion. This indicates a low overall risk of underreporting bias in the dietary assessment instrument (Black 2000). The Goldberg method is an approach to characterise group‐level systematic bias in estimated energy intake derived from dietary assessment tools, but it operates in the absence of objective or biomarker data on total energy expenditure or physical activity (Tooze et al. 2012). Therefore, it does not fully capture the measurement error structure of the 24‐h recall, which is most likely subject to both group‐ and individual‐level systematic biases, as well as random error (Kirkpatrick et al. 2022).

### 2.4 Dietary Modelling Approach and Statistical Analysis

#### 2.4.1 Simulated Fortification Levels

Zambia has implemented a mandatory fortification level, requiring 15 mg of vitamin A per kilogram of table sugar at the point of production. This level was set to provide a margin to allow degradation of roughly one‐third of the vitamin A content. This ensures that table sugar will provide a minimum of 10 mg of vitamin A per kilogram at the consumer level (Fiedler et al. 2013). One population‐level assessment conducted by HarvestPlus in Mkushi District and Nyimba District (Eastern Province) revealed that vitamin A‐fortified sugar collected from households had a median concentration of 8.8 mg/kg (Hotz et al. 2012). It was reported that table sugar sold at retail outlets in the community where our trial was based had a median fortification level of 3.1 mg/kg (25th to 75th percentiles: 1.8–5.5 mg/kg) (Greene et al. 2017). For this analysis, we simulated the impact of five fortification levels of sugar with vitamin A (no sugar fortification, 3.1, 8.8, 10 and 15 mg/kg) on the prevalence of inadequate vitamin A intake (defined as < 900 µg RAE/day [EAR]) and retinol intake > 3000 µg/day (UL) among lactating women.

#### 2.4.2 Estimation of Usual Intake Distributions

We applied the univariate National Cancer Institute (NCI) method to estimate the existing distributions of usual table sugar intake, of usual vitamin A intake from all sources and of usual retinol intake (National Cancer Institute 2020c; Tooze et al. 2010). The NCI method adjusts for within‐person random variation observed in repeated 24‐h recall data, so we make the assumption that the distribution of long‐term average 24‐h recalls is an acceptable proxy for the distribution of usual intake (Kirkpatrick et al. 2022). The NCI models used to estimate usual intake distributions included the following covariates: socio‐economic status (SES), age, alpha (1)‐acid glycoprotein (AGP), C‐reactive protein (CRP), body mass index (BMI), education, weekend, season and sequence of dietary recalls (i.e., first interview vs. subsequent interview). The inflammation biomarkers (i.e., AGP and CRP) were included as covariates as indicators of infection, which might interfere with appetite. The SES score was constructed from the first principal component for the following set of variables: home ownership, number of rooms, drinking water source, house construction material (roof, wall and floor), latrine type, livestock ownership (goat, poultry), access to electricity, solar power ownership, access to media (radio and Television), cellphone ownership, motorcycle ownership and bicycle ownership. The SES score is used to efficiently capture as much socio‐economic information as possible within the studied population using a single variable, although it is not the primary focus of the analysis.

Because less than 5% of the 24‐h recalls had zero intake of vitamin A, the amount‐only NCI model (Davis et al. 2019) was used for vitamin A. Retinol and table sugar were episodically consumed by lactating women included in this analysis (> 10% person‐days with 0 values), which required the use of the two‐part NCI model. The two‐part NCI model jointly estimates the distributions of the probability to consume on a given day and the usual amount consumed on consumption days (National Cancer Institute 2019, 2020b) to estimate the distribution of usual intake. Specifically, the NCI ‘two‐part correlated model’ was used to allow the probability of consumption on a given day to be correlated with the usual amount consumed.

For each hypothesised fortification scenario, the 24‐h recall intake data were modified to reflect fortification of table sugar by adding the amount of vitamin A that would be provided by the reported intake of table sugar to the vitamin A or retinol intake from all other dietary sources. Then we re‐estimated the population usual intake distributions by applying the NCI method to the modified data. To assess the public health contribution of vitamin A‐fortified sugar, we calculated effective coverage under each fortification scenario. Effective coverage was defined as the prevalence of vitamin A inadequacy without sugar fortification minus the prevalence of vitamin A inadequacy with sugar fortification. The prevalence of inadequate vitamin A intake was determined using the EAR cut‐point method (900 µg RAE/day). We further estimated the prevalence of retinol intake above the UL (3000 µg/day) attributed to sugar fortification for each scenario. We modelled higher doses of sugar fortification with vitamin A as a sensitivity analysis to identify the fortification level that would increase the prevalence of retinol intake above the UL among lactating women. We used the bootstrap method with 200 replicates to estimate standard errors (SEs; National Cancer institute 2020b). We present prevalence of inadequate intake, effective coverage and prevalence of retinol intake above the UL with (SE). We summarised the usual intake using mean (±SE), median, 25th and 75th percentiles.

#### 2.4.3 Regression Calibration

Estimating the association between usual dietary intakes and health outcomes is complicated by measurement error in dietary assessment methods. If ignored, this measurement error can severely bias parameters in outcome regression models. The NCI method supports a two‐step regression calibration approach to adjust for the effects of random within‐person measurement error when evaluating associations between usual dietary intakes and health outcomes using repeated 24‐h recalls (Keogh et al. 2020; Kirkpatrick et al. 2022; Shaw et al. 2020). The first step involves fitting a measurement error model to 24‐h recalls and using the fitted model to compute the conditional expectation, or prediction, of usual intake for each respondent, given all the covariates in the disease model. The measurement error model should include other covariates that influence usual intake, even if those covariates do not influence the disease outcome (see recommendations in Shaw et al. 2018; Zhang, Krebs‐Smith, et al. 2011; Zhang, Midthune, et al. 2011). The second step is to use the predictions as an independent variable in the health outcome model. Under the assumption that 24‐h measure usual intake with only random error, the two‐step approach yields consistent (approximately consistent for most nonlinear models) estimates of the parameters in outcome regression models (Kipnis et al. 2009), and the coefficient associated with the computed conditional expectation is taken to be the coefficient for usual intake, even though the conditional expectations themselves are generally poor estimates of the usual intake for particular individuals.

In this study, we used the multivariate NCI method (National Cancer Institute 2020a, 2020b) to jointly model two dietary components: sugar intake and vitamin A intake from nonsugar sources. Covariates common to the measurement error and outcome models were selected based on reported association with vitamin A intake or vitamin A status in previous studies (Baytekus et al. 2019; Cordeiro et al. 2018; Harding et al. 2018; Rubin et al. 2017; Williams et al. 2011), namely: age, SES, educational status, BMI, CRP, AGP and, for breast milk retinol analyses only, breast milk fat. Season was included in the measurement error models to help predict dietary intake but was not included in the outcome models. Season is classified as late post‐harvest (August–October), early lean (November–January), late lean (February–April) and harvest (May–July).

#### 2.4.4 Outcome Regression Models

We first fit a linear regression model with log‐transformed plasma retinol concentration (μmol/L) as a continuous outcome and the usual intake predictions for sugar and vitamin A from nonsugar sources as primary exposures, controlling for age, SES, educational status, BMI, CRP and AGP. Then we fit a logistic regression model where the outcome was log odds of having low plasma retinol (< 1.05 μmol/L), with the same primary exposures and covariates. We also fit a linear regression with log‐transformed breast milk retinol (μmol/L) as a continuous outcome, with breast milk fat as an additional covariate. Lastly, we fit a logistic regression model where the outcome was log odds of having low breast milk retinol (< 1.05 μmol/L), with the same primary exposures as the linear model for breast milk retinol. For each model, we obtained the regression coefficients (*β*) from the original sample and calculated 95% confidence intervals from bootstrap‐generated SEs.

### 2.5 Ethical Statement

The protocol of the trial was reviewed and approved by the Institutional Review Boards at Eres Converge in Zambia, the Johns Hopkins Bloomberg School of Public Health and the University of California, Davis. We obtained authorisation from the Ministry of Health for this research.

## 3 Results

The mean age (±SD) of the study participants was 27.3 (±6.3) years, and the majority were married. About 48% of the lactating women who participated in this study had at least some primary education. The mean (±SD) of haemoglobin was 13.1 (±1.5) g/dL. About 23% of lactating women were underweight (< 18.5 kg/m^2^). The prevalence of low plasma retinol and low breastmilk retinol was 28% (±3.1%) and 23.6% (±3.0%), respectively (Table 1).

**Table 1:** Socio‐demographic characteristics and nutritional status of lactating women in Mkushi, Zambia, enrolled in provitamin A carotenoid‐biofortified maize or retinyl palmitate fortified maize intervention trial, 2016 (*n* = 243).

Variables		*n* (%) or geometric mean (SE)
Maternal education	Attend at least some primary education	112 (47.7)
Maternal occupation	Unpaid family labour	156 (64.2)
	Self‐employed/running a business	66 (27.2)
	Other^a^	21 (8.6)
Marital status	Married	195 (80.3)
Body mass index	< 18.5 kg/m^2^	55 (22.6)
	18.5–24.9 kg/m^2^	140 (57.6)
	≥ 25 kg/m^2^	48 (19.8)
Plasma retinol (μmol/L)		1.22 (0.02)
Breast milk retinol (μmol/L)		1.58 (0.04)
Breast milk retinol per gram of fat (nmol/g fat)		47.83 (0.03)

^a^ Others include farming, farm labour, student, salaried employee, wage management and piecework.

### 3.1 Major Sources of Vitamin A

Fortified sugar and provitamin A‐containing vegetables were the largest contributors to total vitamin A intake (Table S1). The contribution of animal‐source foods (egg, small fish, large fish and liver combined) to total vitamin A intake was about 21%. About 18% of total vitamin A intake came from fortified table sugar, assuming a fortification level 8.8 mg/kg (Hotz et al. 2012). Among lactating women who had consumed table sugar on the previous day, the median (p25, p75) table sugar intake was 35.3 g (29.0, 47.6).

### 3.2 Contribution of Vitamin A‐Fortified Table Sugar to Vitamin A Intake and Dietary Adequacy

The median (p25, p75) usual daily vitamin A intake from foods other than table sugar was 557 (371, 742) µg RAE/day. The median (p25, p75) usual daily vitamin A intake was predicted to increase to 859 (681, 1003) µg RAE with vitamin A fortification of table sugar at 10 mg/kg (the standard in Zambia for consumer‐level samples). The prevalence (±SE) of inadequate vitamin A intake, without vitamin A from fortified table sugar among lactating women, was 83.4% ± 5.6%. The prevalence of inadequate vitamin A intake is predicted to decrease to 76% ± 7.9% with a fortification level of 3.1 mg/kg and to 54.0% ± 13.9% with a fortification level of 10 mg/kg (Table 2, Figure S1).

**Table 2:** Predicted impact of vitamin A‐fortified sugar on usual intake distribution and prevalence of inadequate vitamin A intake among lactating women in Mkushi, Zambia, enrolled in provitamin A carotenoid‐biofortified maize or retinyl palmitate fortified maize intervention trial, 2016 (*n* = 243).

	Total vitamin A intake^a^		Retinol intake^a^
Vitamin A fortification levels in sugar	Median (p25, p75) (µg RAE/day)	Inadequate intake (% ± SE)	Median (p25, p75) (µg retinol/day)
No fortification	557 (371, 742)	83.4 ± 5.6	117 (65, 181)
3.1 mg/kg	643 (442, 839)	76.4 ± 7.9	187 (123, 252)
8.8 mg/kg	828 (657, 967)	59.7 ± 12.9	315 (199, 434)
10 mg/kg	859 (681, 1003)	54.0 ± 13.9	341 (214, 472)
15 mg/kg	996 (788, 1164)	36.6 ± 17.2	454 (274, 644)

Abbreviations: p25, 25th percentile; p75, 75th percentile; SE, standard error.

^a^ Usual intake distributions estimated by the NCI amount model. Retinol intake above 3000 µg retinol is zero at a fortification of 15 mg/kg and lower.

The median retinol intake, excluding retinol intake from fortified table sugar, was 117 (65, 181) µg/day and was predicted to increase to 454 (274, 644) µg/day at a fortification level of 15 mg/kg (Table 2). The 95th percentile of retinol intake at the fortification level of 15 mg/kg was 1050 µg/day. Table sugar fortification with vitamin A in the form of retinol at a fortification level of 15 mg/kg did not increase the prevalence of retinol intake above the UL (3000 µg/day) among lactating women (~ 0%). A sensitivity analysis showed that long‐term average table sugar fortification levels above 40 mg/kg (in the form of retinol) could potentially increase the prevalence of retinol intake above the UL (3000 µg/day) among lactating women (Figure S2).

### 3.3 Association Between Usual Table Sugar Intake and Vitamin A Status

The multivariable regression models showed no significant associations of usual table sugar intake or usual vitamin A intake from nonsugar sources with plasma retinol, low plasma retinol, breast milk retinol or low breast milk retinol (*p*‐value > 0.05) (Table 3). Note that the confidence intervals in Table 3 are not constructed with an adjustment for multiple testing—if they had been, they would have been substantially wider.

**Table 3:** Association of vitamin A status (plasma and breast milk retinol concentration) with usual vitamin A and usual table sugar intakes among lactating women in Mkushi, Zambia, enrolled in provitamin A carotenoid‐biofortified maize or retinyl palmitate fortified maize intervention trial, 2016 (*n* = 243).

Variables	Plasma retinol		Breast milk retinol	
	Log plasma retinol (µmol/L)	Low plasma retinol (< 1.05 µmol/L)	Log breast milk retinol (µmol/L)	Low breast milk retinol (< 1.05 µmol/L)
	*β* (95% CI)	*β* (95% CI)	*β* (95% CI)	*β* (95% CI)
Usual table sugar intake, 10 g/day	0.004 (−0.02, 0.01)	−0.09 (−0.26, 0.08)	0.02 (−0.01, 0.005)	−0.19 (−0.41, 0.03)
Usual vitamin A intake,^a^ 100 µg RAE/day	−0.0002 (−0.01, 0.01)	−0.004 (−0.1, 0.1)	−0.01 (−0.03, 0.004)	0.03 (−0.1, 0.2)
Attend at least primary education (yes)	−0.051 (−0.151, 0.048)	0.946 (−0.226, 2.119)	−0.107 (−0.26, 0.047)	0.567 (−0.609, 1.742)
BMI (kg/m^2^)^b^	0.035 (0.023, 0.048)	−0.394 (−0.612, −0.176)	0.015 (−0.009, 0.038)	0.055 (−0.086, 0.196)
Age (years)	0 (−0.006, 0.007)	0.05 (−0.029, 0.129)	0.002 (−0.009, 0.013)	0.051 (−0.021, 0.123)
Socio‐economic status score	0.052 (0.002, 0.101)	−0.598 (−1.192, −0.005)	0.046 (−0.045, 0.137)	−0.252 (−0.891, 0.387)
Log‐CRP (mg/L)^b^	−0.039 (−0.077, 0)	0.385 (−0.032, 0.803)	−0.011 (−0.09, 0.069)	0.25 (−0.218, 0.719)
Log AGP (g/L)^b^	−0.004 (−0.16, 0.151)	0.003 (−1.281, 1.286)	0.044 (−0.183, 0.271)	−0.125 (−1.651, 1.401)
Milk fat (g)^c^	NA	NA	0.232 (0.166, 0.297)	−1.312 (−2.068, −0.557)

^a^ Usual vitamin A intake from non‐table sugar sources.

^b^ AGP, α_1_‐acid glycoprotein; BMI, body mass index; CRP, C‐reactive protein.

^c^ Milk fat square root was included as a covariate in the breast milk retinol models only.

## 4 Discussion

This study showed that the prevalence of inadequate vitamin A intake is very high among lactating women in Mkushi, Zambia, which is consistent with observed low breast milk vitamin A concentrations (Palmer et al. 2016). Our modelling work predicted that table sugar fortification at the currently mandated fortification level (10 mg/kg) has the potential to reduce the prevalence of dietary vitamin A inadequacy substantially in this population but could not eliminate vitamin A inadequacy at the observed levels of table sugar consumption. At fortification levels below the current standard, the impact is likely to be limited. The potential risk of retinol intake above the UL from table sugar fortification is unlikely among lactating women, even at fortification levels above the current standard. Consistent with the modelled impacts on dietary inadequacy, we did not detect any statistically significant association between sugar intake and vitamin A biomarkers (plasma and breast milk retinol).

Our observations of low vitamin A intake among lactating women are consistent with other studies from Zambia. For example, a dietary survey study from rural villages in eastern Zambia showed that almost all lactating women had vitamin A intake below the EAR (Kaliwile et al. 2019). However, there are limited data available on the patterns of table sugar consumption and the vitamin A levels in table sugar, which together would determine the contribution of the programme to dietary vitamin A intake. A qualitative study found that in Zambia, table sugar is added to tea, traditional beverages, porridge and water (locally referred to as zigolo; Kaliwile et al. 2019). In this study, approximately 50% of lactating women consumed any fortified and/or unfortified table sugar on the previous day, whereas a study conducted in eastern part of Zambia reported that about 76% of women did not have access to or chose not to consume fortified table sugar, suggesting that the majority of the women may not consume sufficient amounts of fortified sugar to achieve adequate vitamin A intake (Kaliwile et al. 2019).

With regard to fortification levels, available data suggest that vitamin A levels in table sugar at the community level are below national targets (Hotz et al. 2012). In the same community and the same year where this study was conducted, only 11% of table sugar samples in markets met the minimum legal requirement fortification level, and about 75% of the table sugar in market samples had a fortification level below 5.5 mg/kg (Greene et al. 2017). The level of vitamin A in the sugar in market samples from Mkushi ranged from 0.2 to 29.9 mg/kg with a median fortification level of 3.1 mg/kg. Taking the median fortification level (3.1 mg/kg) reported from Mkushi as the typical concentration of vitamin A in table sugar, our modelling suggested that table sugar fortification with vitamin A would reduce the prevalence of inadequate vitamin A intake by only 7 percentage points; a very small reduction against a prevalence of 83% in the absence of table sugar fortification. If the fortification level of 10 mg/kg is achieved at the household level, our modelling showed that the prevalence of inadequate vitamin A intake was predicted to be reduced by 30 percentage points. This means that if 100% of the table sugar available in the market meets the standard fortification of 10 mg/kg, still over half of lactating women in this community would have inadequate vitamin A intake. To get the full benefit of the current national table sugar fortification programme in Zambia, adequate monitoring to maintain the standard fortification level is required, and alternative strategies may be necessary to reach women who do not consume table sugar frequently or in sufficient amounts to meet their vitamin A intake requirement.

Our modelling predictions showed that the risk of retinol intake above the UL from fortified sugar was close to zero even if the household fortification level were increased to 15 mg/kg. This is because the retinol intake from all other dietary sources among lactating women is very low and accounts for only 21% of the total vitamin A intake. This study was conducted in a small town in rural Zambia, which might not reflect the risk of retinol intake above the UL among lactating women in urban Zambia. A simulation modelling study from Cameroon showed that scenarios that included urban areas, children and fortification of multiple food vehicles were most likely to result in intake that would exceed the UL (Engle‐Stone et al. 2019). Thus, increasing the fortification level of vitamin A in table sugar in Zambia should be done carefully so as not to increase the risk of retinol intake above the UL for other population groups such as for young children given the fact that high liver vitamin A stores among young children have been reported in Zambia (Mondloch et al. 2015; Tanumihardjo et al. 2015).

We evaluated the association between usual table sugar intake and vitamin A status (measured by plasma retinol and breast milk retinol), hypothesising that such a relationship might be observed if vitamin A‐fortified table sugar were an important source of dietary vitamin A. Usual sugar intake was not significantly associated with either plasma or breast milk retinol levels. This could be because the fortification level of vitamin A in table sugar is low (Greene et al. 2017). Our modelling suggests that table sugar fortification at a lower fortification level has a limited impact on the prevalence of inadequate vitamin A intake. In this sample, the mean usual intake of table sugar fortified at 3.1 or 10 mg/kg would provide only 8.3% or 24.6% of the EAR, respectively. An evaluation study in Cameroon found no increase in plasma retinol‐binding protein or breast milk retinol among women after a year of cooking oil fortification with vitamin A at levels ~ 44% of target (Engle‐Stone et al. 2017). As noted earlier, studies from Latin America showed that sugar fortification programmes implemented according to the standard were successful in increasing vitamin A status.

Usual vitamin A intake from nonsugar sources was not associated with plasma retinol or breast milk retinol concentration. Several studies also failed to find a significant association between vitamin A intake and vitamin A biomarkers, particularly with plasma retinol concentration (Hombali et al. 2019; Booth et al. 1997; Carmem‐Costa‐do‐Nascimento et al. 2011; Meneses and Trugo 2005; Rohner et al. 2016). This may reflect the homoeostatic control of serum retinol over a wide range of liver reserves. Intervention trials showed a significant increase in breast milk retinol concentration but not in plasma retinol concentration following consumption of vitamin A‐rich products (supplement or fortified foods) (Muslimatun et al. 2001; Palmer et al. 2021; Turner et al. 2013). This could be because the concentration of retinol in breast milk via chylomicrons is less likely to be regulated compared to its release into the plasma by the liver (Green et al. 2001; A. C. Ross et al. 2004). Animal‐based studies showed that dietary vitamin A intake was the primary source of vitamin A delivered to the mammary gland for milk vitamin A secretion (Green et al. 2001; Li et al. 2021). However, several studies on lactating women with varying baseline nutritional statuses have demonstrated a weak to non‐significant correlation between dietary vitamin A intake and breast milk retinol levels across different geographic settings (Engle‐Stone et al. 2014; Nimmannun et al. 2022; Olafsdottir et al. 2001).

The absence of association between dietary vitamin A intake and breast milk retinol in this study could be explained by measurement error in dietary assessment, dietary vitamin A intake being too low and/or not variable enough within the study population and/or by the homoeostatic control of retinol coming from maternal stores. The implication that there is no association of vitamin A intake and sugar intake with vitamin A biomarkers (plasma and breast milk retinol concentrations) should be interpreted with caution. This might not necessarily suggest a limited potential or impact of table sugar fortification in Zambia. Concerns have been raised regarding the use of plasma retinol concentration for evaluating fortification programmes, especially in the absence of pre‐post intervention data to compare the plasma retinol distribution (Palmer et al. 2012).

The major strength of this study is that we applied recommended practices for using 24‐h recall to estimate usual intake distributions and assess potential relationships between diet and biomarkers via the use of the NCI method modelling framework. In the absence of detailed data on the quality of implementation of the sugar fortification programme, this kind of modelling study provides insights about the potential contribution of vitamin A‐fortified sugar to usual intake of vitamin A, which might lead to rethinking of the fortification programme and the relevance of other alternative vitamin A interventions. However, the findings of this study should be interpreted with caution. The data for this study were from the baseline survey of a randomised controlled trial from one town in Zambia, which might not be representative of the national table sugar fortification programme or intake of other sources of vitamin A in Zambia. This study was cross‐sectional, which makes it impossible to do a before‐and‐after fortification comparison of the distribution of vitamin A biomarkers.

In this study, we did not measure the actual level of vitamin A in table sugar consumed among women; we assumed that all table sugar in the market is fortified. The fortification levels we simulated might not represent the level of fortification in each of the brands of table sugar consumed in the community. In this community, common practice is to buy small amounts of sugar for immediate use, rather than store a large quantity of sugar at home. So, market table sugar is highly likely to be reflective of what women are actually consuming. We used effective coverage (defined as a change in prevalence of intake below the EAR) as the primary measure of benefit, which does not account for the potential benefits of providing additional vitamin A in amounts that are not large enough to reach the EAR. The potential benefit of small increases in vitamin A intake that may lead to improvement in vitamin A biomarkers is difficult to quantify. In this study, information on liver vitamin A stores was not available; this biomarker may be more likely to vary in relation to dietary intake compared to plasma retinol, which is under homoeostatic control. Although no association was observed between table sugar intake and plasma or breast milk retinol, there may be benefits to the national programme that were not captured in this study population.

The primary weakness of this analysis (shared with any analysis of nutritional data not obtained from one of the few accepted recovery biomarkers) is that the effects of measurement error are only partially mitigated by the statistical methods employed herein. Systematic biases at the group and individual level may distort the shape of the estimated usual intake distributions as well as the magnitude of the bias in estimated outcome model parameters. Furthermore, although the use of regression calibration adjusts for bias due to random error in outcome model parameters (while leaving bias due to systematic errors unaddressed), the power to detect a significant relationship is reduced compared to what it would have been if usual intake could have been directly and exactly measured. This fact may have contributed to the null findings concerning retinol concentration and vitamin A or sugar intake.

In conclusion, our analysis suggests that vitamin A‐fortified table sugar has the potential to substantially reduce, but not eliminate, the high prevalence of inadequate vitamin A intake in this population. We found no evidence of impact of consuming table sugar on vitamin A status biomarkers in this study population, although this does not exclude the possibility of benefits to other population groups or communities. Ensuring adequate fortification by monitoring the quality of the fortification programme is necessary to improve micronutrient consumption. Other complementary vitamin A interventions, in addition to efforts to enforce the table sugar fortification mandate, are likely required to reduce vitamin A deficiency among lactating women in Zambia.

## Author Contributions

Conceptualisation: D.H., R.E.‐S. and A.C.P. Methodology: D.H., R.E.‐S., A.C.P., B.C., H.L., K.W.D., C.D.A., M.J., M.G., M.C. and M.J.H. Data analysis: D.H., R.E.‐S., H.L., C.D.A., B.C. and K.W.D. Writing – original draft preparation: D.H. and R.E.‐S. Writing – review and editing: R.E.‐S., A.C.P., B.C., H.L., K.W.D., C.D.A., M.J., M.G., M.C. and M.J.H. All authors have read and agreed to the published version of the manuscript.

## Disclosure

The views expressed do not necessarily reflect those of HarvestPlus or other funders of this study.

## Conflicts of Interest

The authors declare no conflicts of interest.

## Supporting information


**Supporting Table 1:** Consumption of vitamin A rich foods and mean contribution to total vitamin A intake among lactating women in Mkushi, Zambia enrolled in provitamin A carotenoid‐biofortified maize or retinyl palmitate fortified maize intervention trial, 2016 (n = 243).
**Supporting Figure 1:** Predicted impact of vitamin A‐fortified table sugar on usual vitamin A intake distribution among lactating women in Mkushi, Zambia. The purple broken vertical line indicates the EAR (900 µg RAE/d).
**Supporting Figure 2:** Predicted impact of vitamin A‐fortified sugar at various fortification levels on usual retinol intake distribution among lactating women in Mkushi, Zambia. The purple broken vertical line indicates the UL (3000 µg retinol/d).

## Data Availability

The data that support the findings of this study are available from the corresponding author upon reasonable request.
