# Supplementary material for: Simulated Impact of Vitamin A‐Fortified Sugar on Dietary Adequacy and Association of Usual Sugar Intake With Plasma and Breast Milk Retinol Among Lactating Zambian Women
Source: Matern Child Nutr. 2025 Aug 6;21(4):e70077. doi: 10.1111/mcn.70077 (PMC12454194; doi:10.1111/mcn.70077)
Supplement: Supplementary file 1 — Supporting Table 1: Consumption of vitamin A rich foods and mean contribution to total vitamin A intake among lactating women in Mkushi, Zambia enrolled in provitamin A carotenoid‐biofortified maize or retinyl palmitate fortified maize intervention trial, 2016 (n = 243). Supporting Figure 1: Predicted impact of vitamin A‐fortified table sugar on usual vitamin A intake distribution among lactating women in Mkushi, Zambia. The purple broken vertical line indicates the EAR (900 µg RAE/d). Supporting Figure 2: Predicted impact of vitamin A‐fortified sugar at various fortification levels on usual retinol intake distribution among lactating women in Mkushi, Zambia. The purple broken vertical line indicates the UL (3000 µg retinol/d). [file MCN-21-e70077-s001.docx]

**Supplementary files**

**Supplemental Table 1: Consumption of vitamin A rich foods and mean contribution to total vitamin A intake among lactating women in Mkushi, Zambia enrolled in provitamin A carotenoid-biofortified maize or retinyl palmitate fortified maize intervention trial, 2016 (n= 243).**

| Food | Number of servings per day (Median (p25, p75)) | Quantity consumed per serving (g raw weight)  (Median (p25, p75)) | Mean percent of total vitamin A intake  (mean ± SD) |
| --- | --- | --- | --- |
| Sugar^#^ | 1(0, 1) | 31(22, 39) | 18 ± 12.3 |
| Rape leaves | 0(0, 1) | 22(20, 39) | 15.7 ± 11.1 |
| Sweet potato | 0(0, 1) | 210(144, 210) | 12.2 ± 12 |
| Other veg^‡^ | 0(0, 0) | 144(84, 210) | 7.6 ± 11.1 |
| Eggs | 0(0, 0) | 69(50, 90) | 6.1 ± 5.3 |
| Butter or margarine | 0(0, 0) | 22(20, 27) | 5.8 ± 5.7 |
| Small fish | 0(0, 1) | 33(20, 38) | 5.8 ± 11.3 |
| Other DGLV^*^ | 0(0, 0) | 29(24, 47) | 5 ± 8.1 |
| Mango | 0(0, 0) | 340(170, 340) | 4.7 ± 6.8 |
| Pumpkin leaves | 0(0, 0) | 47(24, 52) | 3.3 ± 5.9 |
| Tomato | 4(3, 4) | 32(19, 44) | 2.6 ± 2.1 |
| Other fruit^**^ | 0(0, 0) | 140(110, 165) | 1.8 ± 3.4 |
| Other large fish^***^ | 0(0, 0) | 52(33, 72) | 1.7 ± 3.3 |
| Cabbage | 0(0, 0) | 23(20, 43) | 1.6 ± 3.5 |
| Liver/offals | 0(0, 0) | 15(11, 22) | 1.4 ± 5.9 |

Note: Only foods contributing, on average, at least 1% of total intake are listed in the table

^#^ assumed sugar fortification level was 8.8 mg/kg

^*^Any leafy vegetables other than rape leaves, cabbage or pumpkin leaves

^**^Any fruit other than mango, banana or citrus fruit

‡Any vegetables other than leafy vegetables, root vegetables, tomato, eggplant, mushrooms, okra

^***^Any large fish other than tilapia species


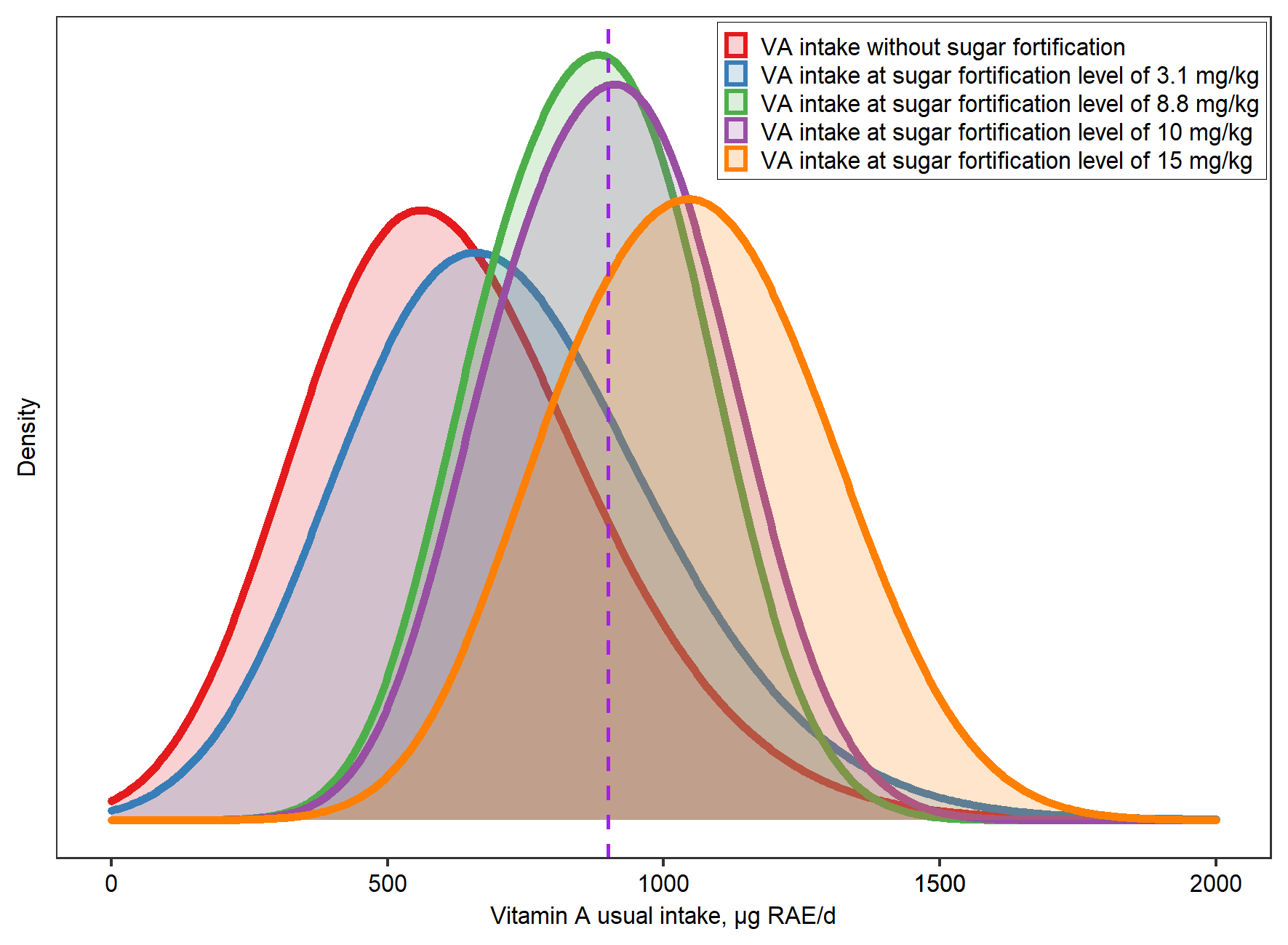


**Supplemental Figure 1: Predicted impact of vitamin A-fortified table sugar on usual vitamin A intake distribution among lactating women in Mkushi, Zambia. The purple broken vertical line indicates the EAR (900 µg RAE/d).**


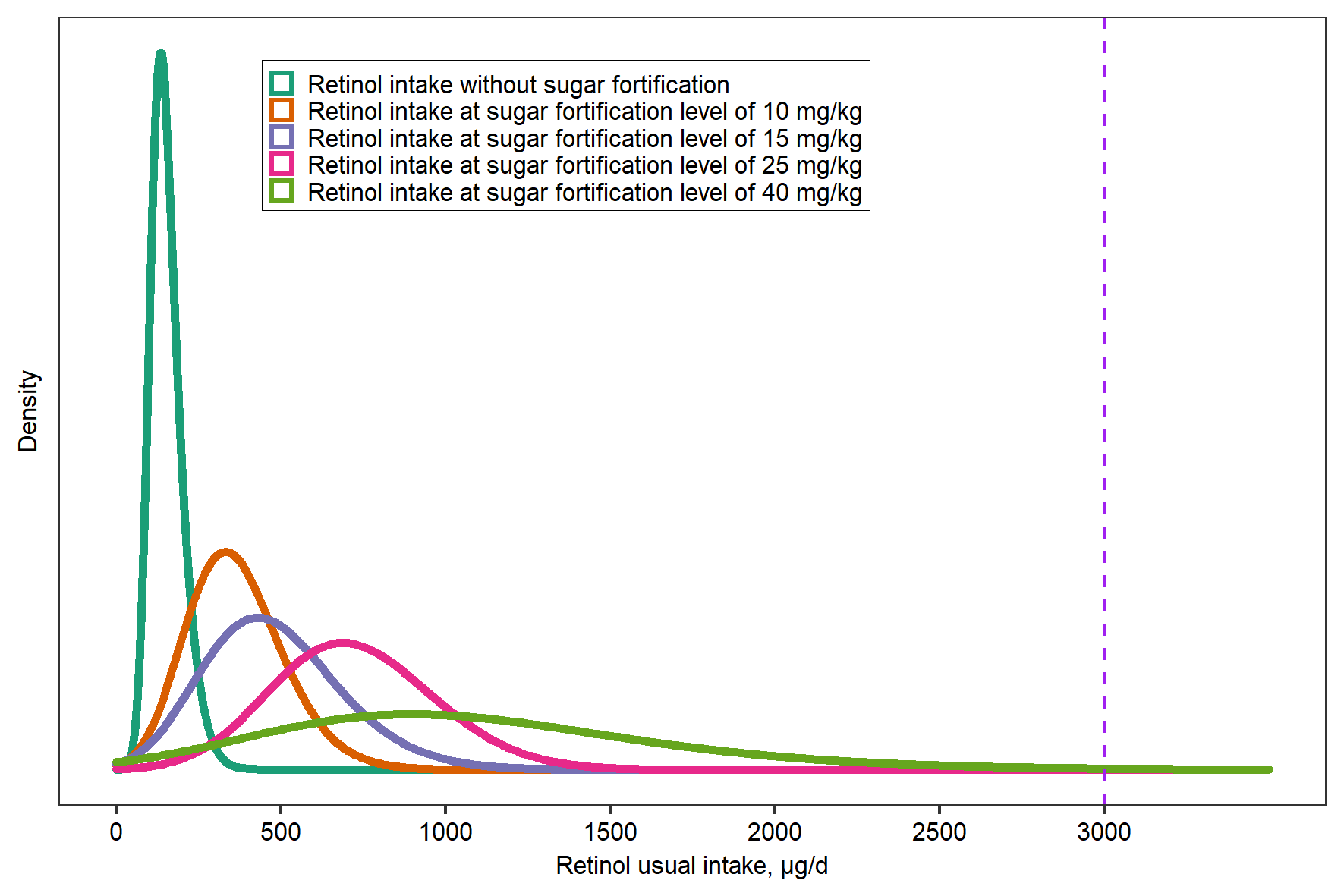


**Supplemental Figure 2: Predicted impact of vitamin A-fortified sugar at various fortification levels on usual retinol intake distribution among lactating women in Mkushi, Zambia. The purple broken vertical line indicates the UL (3000 µg retinol/d).**
